# Fractures reduction with osteoporotic treatments in patients over 75-year-old: A systematic review and meta-analysis

**DOI:** 10.3389/fragi.2022.845886

**Published:** 2022-11-02

**Authors:** Michel Guillaumin, Bastien Poirson, Aurélie Gerazime, Marc Puyraveau, Thomas Tannou, Fréderic Mauny, Éric Toussirot

**Affiliations:** ^1^ Département de Gériatrie, CHU, Besançon, France; ^2^ Unité de Méthodologie, INSERM CIC-1431 Centre Investigation Clinique CHU, Besançon, France; ^3^ INSERM CIC-1431 Centre d’Investigation Clinique, CHU, Besançon, France; ^4^ INSERM CIC-1431 Centre Investigation Clinique et Département de Rhumatologie, CHU, Besançon, France

**Keywords:** osteoporosis treatments, osteoporosis, fractures, older people, systematic review

## Abstract

**Background:** Osteoporosis consists in the reduction of bone mineral density and increased risk of fracture. Age is a risk factor for osteoporosis. Although many treatments are available for osteoporosis, there is limited data regarding their efficacy in older people.

**Objective:** To evaluate the efficacy of osteoporosis treatments in patients over 75 years old.

**Methods:** We reviewed all published studies in MEDLINE, Cochrane and EMBASE including patients over 75 years old, treated by osteoporosis drugs, and focused on vertebral fractures or hip fractures.

**Results:** We identified 4,393 records for review; 4,216 were excluded after title/abstract review. After full text review, 19 records were included in the systematic review. Most studies showed a reduction in vertebral fracture with osteoporosis treatments, but non-significant results were observed for hip fractures. Meta-analysis of 10 studies showed that lack of treatment was significantly associated with an increased risk of vertebral fractures at one (OR = 3.67; 95%CI = 2.50–5.38) and 3 years (OR = 2.19; 95%CI = 1.44–3.34), and for hip fractures at one (OR = 2.14; 95%CI = 1.09–4.22) and 3 years (OR = 1.31, 95%CI = 1.12–1.53).

**Conclusion:** A reduction in the risk of vertebral fractures with osteoporosis treatments was observed in most of the studies included and meta-analysis showed that lack of treatment was significantly associated with an increased risk of vertebral fractures. Concerning hip fractures, majority of included studies did not show a significant reduction in the occurrence of hip fractures with osteoporotic treatments, but meta-analysis showed an increased risk of hip fractures without osteoporotic treatment. However, most of the data derived from *post hoc* and preplanned analyses or observational studies.

## Introduction

Osteoporosis is a skeletal systemic disease characterized by a reduction in bone mass and bone mineral density (BMD), deterioration of the bone micro-architecture, and a subsequent increase in the risk of fracture of the spine, hip and other sites (Kanis et al., 2013; Cosman et al., 2014; Black and Rosen, 2016).

Worldwide, osteoporosis causes 8.9 million fractures each year, with one fracture occurring approximately every 3 s (Johnell and Kanis, 2006). By 2025, osteoporosis fractures and costs are projected to grow by >48% to >3 million fractures (Burge et al., 2007).

Fractures are associated with a high mortality rate and have a significant influence on the quality of life of patients with osteoporosis (Cooper et al., 1993; Lips and van Schoor, 2005; Sakamoto et al., 2006), sometimes leading to a need for long-term nursing care and a loss of healthy life expectancy (Tajeu et al., 2014).

Many risk factors have been established for osteoporosis, including age (Hui et al., 1988; Ross, 1996; De Laet et al., 1998; Sanders et al., 1999; Jackson and Mysiw, 2014). The incidence of osteoporotic fractures increases with advancing age: vertebral fractures are the most common, with a prevalence of approximately 20% in women aged 75 years, and 40% in women aged 80 years (Grados et al., 2004). Similarly, the cumulative incidence of hip fractures in women aged 80 years is approximately 30% (Cooper et al., 1992).

Available pharmacological therapies for the treatment of postmenopausal osteoporosis include antiresorptive drugs such as bisphosphonates and denosumab, a fully human monoclonal antibody against the receptor activator of nuclear factor-κB ligand; conversely, the parathyroid hormone analog teriparatide has a bone anabolic mechanism. The reduction of fractures with these treatments has been well demonstrated by large randomized placebo-controlled clinical trials (Black et al., 1996; Cummings et al., 1998; Black et al., 2000; Reginster et al., 2000; McClung et al., 2001; Neer et al., 2001; Black et al., 2007; Lyles et al., 2007; Cummings et al., 2009; Kendler et al., 2018). Recently, a meta-analysis performed by Nayak (Nayak and Greenspan, 2017) showed that osteoporosis treatments reduce the risk of vertebral and possibly non-vertebral fractures in men with osteoporosis.

However, despite the high risk of osteoporotic fractures in the geriatric population, most studies have included a limited number of people aged 75 or over.

The aim of this systematic review and meta-analysis was therefore to review the published literature on the efficacy of osteoporosis treatments in reducing the most common fractures in subjects aged over 75 years. We focused on the two most common and serious types of fractures in older people, namely hip (HF) and vertebral fractures (VF).

## Methods

This review was conducted in accordance with the Cochrane Handbook for Systematic Reviews (V6.1) (Higgins et al., 2020) and is reported according to the PRISMA (Preferred Reporting Items for Systematic Reviews and Meta-analysis) statement (Moher et al., 2015). The PICO method (Population, Intervention, Comparison, Outcome) was used before making the literature search to formalize the objective of the study:

1) Population: patients over 75 years old who were receiving osteoporotic treatment.
2) Intervention: taking a single osteoporosis treatment.
3) Comparison: not taking an osteoporotic treatment.
4) Outcomes: development of an osteoporotic fracture.

### Search strategy and selection criteria

We performed an electronic search of Medline, the Cochrane Library and Embase on 3 March 2020, which was also updated on 11 July 2020 and again on 17 August 2020. The search strategy was developed with a research librarian. The keywords used are available in Appendix 1.

We also searched for ongoing clinical trials (in ClinicalTrials.gov) and manually checked bibliography of previously published reviews to identify potentially eligible studies.

The primary outcome was to assess the risk of fractures in subjects older than 75 years who received a single osteoporosis treatment.

We thus included all studies that evaluated the efficacy of osteoporosis treatments in subjects aged over 75 years (at the start of treatment), in terms of the risk of osteoporotic fractures. If the studies did not include only subjects over 75 years, they were only eligible for inclusion if they reported separate data for this age group.

Sub-group analysis or pooled analysis specifically reporting outcomes in our pre-specified age group were included in this systematic review.

Exclusion criteria for the studies were: meta-analysis/systematic reviews, studies including subjects over 75 years but without independent analysis of this age group, studies with patients receiving two or more osteoporosis treatments, studies without a control group (including case reports, descriptive observational studies, etc.) or with a control group under 75 years or also receiving osteoporotic treatment, and studies not written in English or French.

### Study selection and data extraction

Two independents reviewers (MG and BP) examined each title and abstract to identify potentially eligible articles. Records deemed eligible, and records that did not contain enough information to confirm their inclusion, underwent full text review. Disagreements were resolved through discussion, and by a third reviewer (AG, MP or FM) if required.

All data were summarized in a spreadsheet recording the first author, year of publication, country, design, sample and mean age of patients, gender, molecule and dose, medical history, type of fracture, and fracture incidence at different times. Another independent reviewer (AG) verified all data extraction.

Authors and industry sponsors were contacted to obtain more information and clarification of subgroup analyses, or additional data on the relevant age group, where necessary.

### Risk of bias assessment

We excluded studies that were not written in English or French, due to language barrier. In addition, some articles included subjects over 75 years, but the information needed for the systematic review could not be retrieved.

Risk of bias was independently assessed by two reviewers (MG and BP). Studies were judged as either as ‘low risk’, ‘unclear’ or ‘high risk’ according to the Cochrane Handbook for Systematic Reviews of Interventions (Higgins et al., 2011). We considered the methodological quality for each study on the basis of the following categories: selection bias, performance bias, detection bias, potential for attrition bias, potential for reporting bias and other potential bias.

### Statistical analysis

Where suitable statistical summary data were available, we combined selected outcome data in pooled meta-analyses using the Cochrane statistical package RevMan (Cochrane Training, 2022). Odds ratios (ORs) and 95% confidence intervals (CI) were calculated to estimate the fracture rate. Significance was defined as a *p* < 0.05. We assessed statistical heterogeneity using the I^2^ test to determine whether fixed effects (I^2^ < 50%) or random effects (I^2^ ≥ 50%) modelling should be used.

When two studies included an identical population derived from the same randomized controlled trial (RCT), only one study was included in the meta-analysis.

A first sensitivity analysis was carried out in addition to the OR calculations, calculation of the RRs on all the criteria of the HF and VF subgroups, then the inclusion of the cohorts allows a second sensitivity analysis.

## Results

### Study selection

**FIGURE 1:**
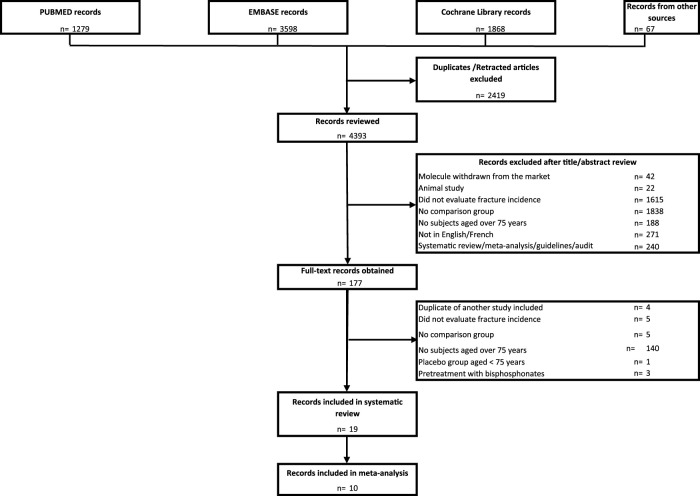
PRISMA flow chart.

The literature search identified 6,812 records for review, of which 2,419 were excluded because they were duplicates, leaving 4,393 unique records for review. A total of 4,216 were excluded after review of the title/abstract. After full text review, 19 records (Ensrud, 1997; Mcclung et al., 2001; Marcus et al., 2003; Boonen et al., 2004; Boonen et al., 2006; McCloskey et al., 2006; Morin et al., 2007; Eastell et al., 2009; Boonen et al., 2010; Boonen et al., 2011; McClung et al., 2012; Nakano et al., 2014; Bawa et al., 2015; Greenspan et al., 2015; Cosman et al., 2016; Axelsson et al., 2017; Cosman et al., 2017; Bergman et al., 2018; McClung et al., 2018) selected independently by MG and BP (100% concordance) were included in the systematic review and 10 (Ensrud, 1997; Mcclung et al., 2001; Marcus et al., 2003; Boonen et al., 2004; McCloskey et al., 2006; Boonen et al., 2010; Boonen et al., 2011; McClung et al., 2012; Axelsson et al., 2017; Bergman et al., 2018) in the meta-analysis (Table 1). Figure 1 shows a flowchart of the literature search and study selection.

**TABLE 1:** Summary of included studies.

Author, years	Country	Selection criteria	Sex	Age	Number of participants	Intervention	Comparison	Primary Outcome(s)	Confirmation of fracture	Main results
**Randomized controlled trial**										
*McClung, 2001 [32]*	*International*	*At least one nonskeletal risk factor for HF, a T score < -4 at FN or a T score < -3 plus a hip-axis length of 11.1 cm or greater*	*W*	*>80 years*	*3,886*	*Risedronate 2.5 mg or 5 mg/d*	*Placebo*	*Incidence of HF*	*Radiography*	*RR = 0.8; 95%CI = 0.6–1.2; p = 0.35*
*McCloskey, 2007 [33]*	*United Kingdom (community-dwelling)*	*Randomly recruited from general practice lists (not necessarily proven osteoporosis or any risk factors for fracture)*	*W*	*>75 years*	*5,592*	*Clodronate 800 mg/d*	*Placebo*	*Incidence of HF*	*Hospital notes, discharge/general practitioner letters, copies of radiographic reports, or review of radiographs*	*During the first year:HR = 1.31; 95%CI = 0.84-2.03During the third year: HR = 0.49; 95%CI = 0.23–1.06*
Greenspan, 2015 [34]	United states (nursing homes or assisted living facility)	T-score<-2 spine, hip or radius or history of VF or HF	W	>65 y	181	Single 5 mg dose of zoledronic acid	Placebo	Change in BMD of the total hip and spine at 12 months*	DXA*	OR = 0.76; 95%CI = 0.25–2.28; *p* = 0.62
Costman, 2016 [35]	International	T score <-2.5 to -3.5 at the total hip or FN	W	>55 y	2,240	Romosozumab 210 mg/m	Placebo	Incidence of VF	Radiography	Data not shown concerning subjects >75 years
**Post hoc analysis**										
*Boonen, 2010 [36]*	*International*	*T-score < -2.5 at FN with or without VFx or T-score < -1.5 at FN with radiological evidence of at least two mild VFx or one moderate VF*								
*OR*										
*90 days after HF*	*M + W*	*>75years*	*3,888*	*Zoledronic acid 5 mg/y*	*Placebo*	*Incidence of clinical VF and nVF and any clinical fracture*	*Radiography*	*At 1 year: HR = 0.39; 95%CI 0.19–0.82; p = 0.09*		
*At 3 years: HR = 0.34; 95%CI = 0.21–0.55; P< 0.001*										
*Ensrud, 1997 [37]*	*United states*	*BMD at the FN of 0.68 g/cm2 or less (approximately Tscore < -2) and at least 1 VF*	*W*	*>75 years*	*539*	*Alendronate 5 mg/d then 10 mg/d*	*Placebo*	*Incidence of VF*	*Radiography*	*RR = 0.62; 95%CI = 0.41–0.94*
*Boonen, 2004 [38]*	*International*	*T-score < -2.5 at FN or at least one VF*	*W*	*>80years*	*1,392*	*Risedronate 5 mg/d*	*Placebo*	*Incidence of VF*	*Radiography*	*After 1 year HR = 0.19; 95%CI = 0.09–0.40; P< 0.001After 3 years HR = 0.56; 95%CI = 0.39–0.81; P< 0.003*
*Boonen, 2011 [39]*	*International*	*T score < −2.5 but > -4.0 at the lumbar spine or total hip*	*W*	*>75 years*	*2,471*	*Denosumab 60 mg/6m*	*Placebo*	*Incidence of VF and HF*	*Radiography*	*Significant reduction in the risk of HFx in subjects aged 75 years or older (2.3% placebo* vs. *0.9% denosumab; p < 0.01)*
*Marcus, 2003 [40]*	*International*	*At least one moderate/two mild VFx or fewer than two moderate VFx and Tscore < -1*	*W*	*>75years*	*1,637 including 285 > 75years*	*Teriparatide 20 µg or 40 μg/d*	*Placebo*	*Relationship between risk of VF/nVF fractures and age*	*Radiography*	*Treatment was associated with a similar reduction in the relative risk of fracture in eachsubgroup of age*
Nakano, 2013 [41]	Japan	Primary osteoporosis with one to five VF and T-score<-1.67 at the lumbar spine, FN, total hip, or distal radius	M + W	>75 y	283	Teriparatide 56.5µg/w	Placebo	Incidence of VF	Radiography	RR = 0.32; 95%CI = 0.13–0.80; *p* = 0.015
*McClung, 2012 [42]*	*International*	*T-score < -2.5 at either the lumbar spine or total hip and >-4.0 at both sites*	*W*	*>75 years*	*2,471*	*Denosumab 60mg/*6 m	*Placebo*	*Incidence of VF*	*Radiography*	*RR = 0.36, 95%CI = 0.25–0.53*
McClung, 2018 [43]	International	At least 2 mild or at least 1 moderate lumbar or thoracic VF or a history of nVF within the preceding 5 years with T-score<-2 at the lumbar spine or hip or without prior fracture but T-score<-3	W	>80 y	94	Abaloparatide 80 μg/d	Placebo	Incidence of VF	Radiography	2 new VFx in placebo group,0 new VFx in Abaloparatide group(not statistically significant)
**Preplanned and post hoc analysis**										
Eastell, 2009 [44]	International	T-score<-2.5 at FN with or without evidence of existing VFx or a T-score<-1.5 at FN with radiological evidence of at least two mild VFx or one moderate VF	W	>75 y	2,949	Zoledronic acid 5 mg/y	Placebo	Incidence of VF, nVF and HF	Radiography	VF incidence (%): 4.8 (Zoledronate) vs 12 (Placebo); *p* < 0.0001
HF incidence (%): 2.1 (Zoledronate) vs 2.7 (Placebo); *p* = 0.3511										
Prespecified subgroup analysis										
Boonen, 2006 [45]	International	At least one moderate/two mild VFx or fewer than two moderate VFx and Tscore<-1	W	>75 y	244	Teriparatide 20 μg/d	Placebo	Incidence of VF	Radiography	RR = 0.35; *p* < 0.05
Costman, 2016 [46]	International	At least 2 mild or at least 1 moderate lumbar or thoracic vertebral fractures or a history of nonvertebral fracture within the preceding 5 years with T-score<-2 at the lumbar spine or hip or without prior fracture but T-score<-3	W	>75 y	248	Abaloparatide 80 μg/d	Placebo	Incidence of VF	Radiography	RR = 0.48; 95%CI = 0.09–2.55
**Prospective cohort**										
*Axelsson, 2017 [47]*	*Sweden*	*Prior HF*	*M + W*	*>80 years*	*9,805*	*Alendronate*	*No treatment*	*Incidence of HF*	*Code for surgical procedure*	*HR per year = 0.91; 95%CI = 0.85–0.97; P< 0.01*
**Retrospective cohort**										
*Bergman, 2018 [48]*	*Sweden*	*History of clinical fracture from 2006 to 2011*	*M + W*	*>50 years*	*83,104 including 22,830 > 80years*	*Alendronate, Risedronate, or Zoledronic acid*	*No treatment*	*Incidence of any clinical fracture and HF*	*ICD-10 codes*	*In adults over 80 years, during the first 6 months, the rate of HF was higher in bisphosphonate users than in non-users. From 6 to 12 months: the rate of HF was similar in users and non-users*
Morin, 2007 [49]	Quebec	Hospitalization for HF between 1996 and 2002	M + W	>65 y	20,644 including 11,573 > 80 y	Etidronate, Alendronate, Risedronate, Raloxifene, Calcitonin or HRT	No treatment	Incidence of HF	ICD-9 codes	In adults over 80 years, HR = 0.92; 95%CI = 0.77–1.10
Bawa, 2015 [50]	United states	Presence of a fragility fracture (wrist, proximal part of the humerus, hip, or vertebral) and prescription medication coverage as a part of insurance	M + W	>50 y	7,502 (number of subjects >80 years not shown)	Bisphosphonates, Teriparatide, Denosumab, Raloxifene or Calcitonin	no treatment	Incidence of new HF, VF, Humerus or Wrist fracture	CPT codes	In adult over 80 years,VF:OR = 0.57; 95%CI = 0.42–0.78; *p* < 0.01HF: OR = 0.81; 95%CI = 0.61–1.07; *p* < 0.01

Abbreviations: HF = hip fracture; FN = femoral neck; cm = centimeter; W = women; y = years; mg = milligrams; d = days; RR = relative risk; CI = confidence interval; HR = hazard ratio; VF = Vertebral Fracture; BMD = bone mineral density; DXA = dual energy x‐ray absorptiometry; OR = odd radio; m = months; VFx = vertebral fractures; μg = microgram; M = men; w = weeks; nVF = nonvertebral Fracture; ICD = International Statistical Classification of Diseases; HRT = Hormone Replacement Therapy; CPT = Current Procedural Terminology.

^∗^ Secondary outcome : incidence of VF mesured by DXA.

In italic: studies included in meta analysis.

### Risk of bias assessment

**FIGURE 2:**
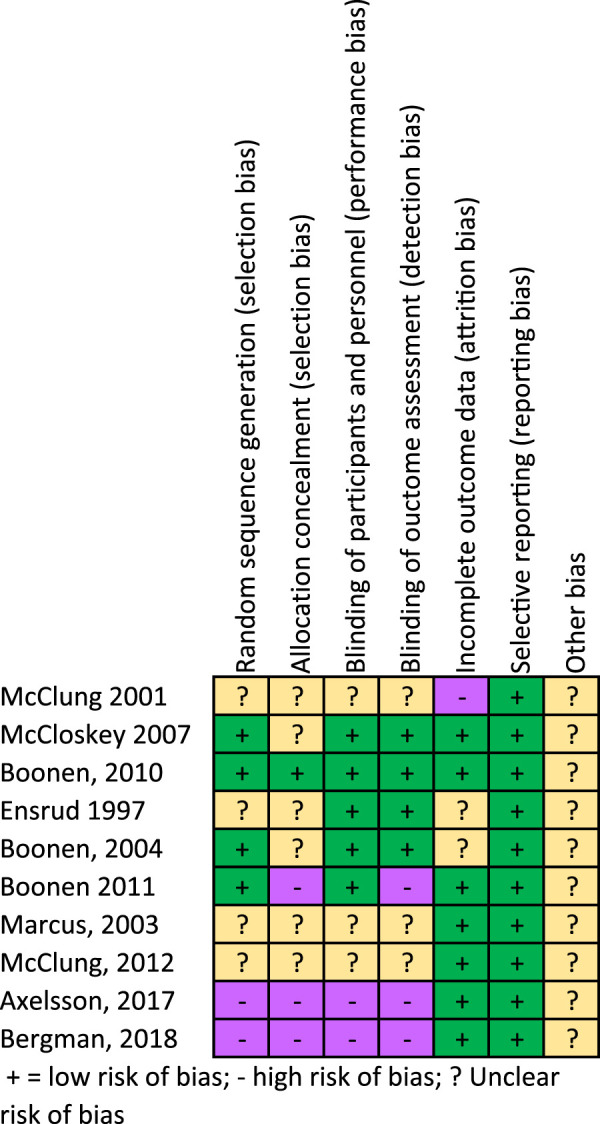
Risk of bias summary.

Risk of bias is summarized for studies included in meta-analysis in Figure 2 *.*

### Study characteristics

Table 1 summarizes the key characteristics of the selected studies in systematic review and meta-analysis.

The included studies had a number of different designs, including four randomized controlled trials (RCTs) (Mcclung et al., 2001; McCloskey et al., 2006; Greenspan et al., 2015; Cosman et al., 2016), eight *post-hoc* analyses (Ensrud, 1997; Marcus et al., 2003; Boonen et al., 2004; Boonen et al., 2010; Boonen et al., 2011; McClung et al., 2012; Nakano et al., 2014; McClung et al., 2018), one pre-planned and *post-hoc* analysis (Eastell et al., 2009), two pre-specified subgroup analyses (Boonen et al., 2006; Cosman et al., 2017), one prospective cohort study (Axelsson et al., 2017) and three retrospective cohort studies (Morin et al., 2007; Bawa et al., 2015; Bergman et al., 2018).

The molecules studied were alendronate, risedronate, zoledronic acid, clodronic acid, etidronate, denosumab, romosozumab, teriparatide, abaloparatide (Table 1). Two retrospective cohorts also studied raloxifene, hormone replacement therapy and calcitonin (Morin et al., 2007; Bawa et al., 2015) but these treatments are not approved for osteoporosis treatment in older people.

Studies were conducted in North America, Europe, Australia, China, or Japan. Included studies were published between 1997 and 2018 and study duration ranged from one to 3 years except for one study lasting 7 years (Axelsson et al., 2017). The majority of studies included only women with postmenopausal osteoporosis (Ensrud, 1997; Mcclung et al., 2001; Marcus et al., 2003; Boonen et al., 2004; Boonen et al., 2006; McCloskey et al., 2006; Eastell et al., 2009; Boonen et al., 2011; McClung et al., 2012; Greenspan et al., 2015; Cosman et al., 2016; Cosman et al., 2017; McClung et al., 2018), and did not include men or patients with secondary causes of osteoporosis. Osteoporosis fractures were detected radiographically in most studies, except for one in which fractures were detected by dual energy x-ray absorptiometry (DXA) (Greenspan et al., 2015). Fracture incidence was the primary endpoint for all studies, except the study by Greenspan, where fracture was a secondary endpoint (Greenspan et al., 2015).

### Effect on vertebral fractures

Thirteen studies evaluated the incidence of VF with osteoporosis treatment (Ensrud, 1997; Marcus et al., 2003; Boonen et al., 2004; Boonen et al., 2006; Eastell et al., 2009; Boonen et al., 2010; McClung et al., 2012; Nakano et al., 2014; Bawa et al., 2015; Greenspan et al., 2015; Cosman et al., 2016; Cosman et al., 2017; McClung et al., 2018). There were only two RCTs, including one with a subgroup of subjects over 75 years, but data concerning this age-group were not given (Cosman et al., 2016), while the second involved subjects over 65 years living in nursing homes, with a mean age of 85 years. The incidence of VF was a secondary outcome in this study, and was not significantly lower in the zoledronic acid group than in the placebo group (OR = 0.76; 95%CI = 0.25–2.28; *p* = 0.62) (Greenspan et al., 2015). One retrospective cohort performed stratification by age and observed a significant reduction in 3-year VF incidence in the subgroup over 80 years (OR = 0.57; 95%CI = 0.42–0.78; *p* < 0.01) (Bawa et al., 2015). All other studies were *post hoc* or prespecified subgroup analyses from RCTs *versus* placebo and concerned subjects over 75 years (Ensrud, 1997; Marcus et al., 2003; Boonen et al., 2004; Boonen et al., 2006; Eastell et al., 2009; Boonen et al., 2010; McClung et al., 2012; Nakano et al., 2014; Cosman et al., 2017; McClung et al., 2018). Among these studies, two did not show significant results (Cosman et al., 2017; McClung et al., 2018). These two studies were derived from the same RCT and concerned abaloparatide (Miller et al., 2016). The remaining studies showed a significant decrease in the incidence of new vertebral fractures in the treatment group.

### Effect on hip fractures

Nine studies (Mcclung et al., 2001; McCloskey et al., 2006; Morin et al., 2007; Eastell et al., 2009; Boonen et al., 2010; Boonen et al., 2011; Bawa et al., 2015; Axelsson et al., 2017; Bergman et al., 2018) evaluated the incidence of HF with osteoporosis treatment. There were two RCTs (Mcclung et al., 2001; McCloskey et al., 2006), including one study that included women older than 80 years (Mcclung et al., 2001). In that study, at 3 years, there was no significant reduction in the risk of HF (RR = 0.8; 95%CI = 0.6–1.2; *p* = 0.35). The second study was a single center study in elderly community-dwelling women older than 75 years (McCloskey et al., 2006). The particularity of this study is that subjects did not necessarily have osteoporosis or an underlying fracture. No significant reduction in the incidence of HF was observed after 1 year (RR = 1.31; 95%CI = 0.6–1.2) or after 3 years (RR = 0.49; 95%CI = 0.23–1.06). Among the three post *hoc* analyses, one study found significant results (Boonen et al., 2011), while the two others did not (Eastell et al., 2009; Boonen et al., 2010). A prospective cohort study by Axelsson showed that alendronate was associated with a reduced risk of HF (HR per year = 0.91; 95%CI = 0.85–0.97; *p* < 0.01) (Axelsson et al., 2017). The three retrospective cohorts selected did not report a significant reduction in HF among patients receiving treatment (Morin et al., 2007; Bawa et al., 2015; Bergman et al., 2018).

### Meta-analysis

Ten studies (Ensrud, 1997; Mcclung et al., 2001; Marcus et al., 2003; Boonen et al., 2004; McCloskey et al., 2006; Boonen et al., 2010; Boonen et al., 2011; McClung et al., 2012; Axelsson et al., 2017; Bergman et al., 2018) reported sufficient data for separate meta-analysis for the following drugs: alendronate, risedronate, zoledronic acid, denosumab and clodronate for VF; and alendronate, risedronate, zoledronic acid, denosumab and teriparatide for HF.

#### Vertebral fracture

**FIGURE 3:**
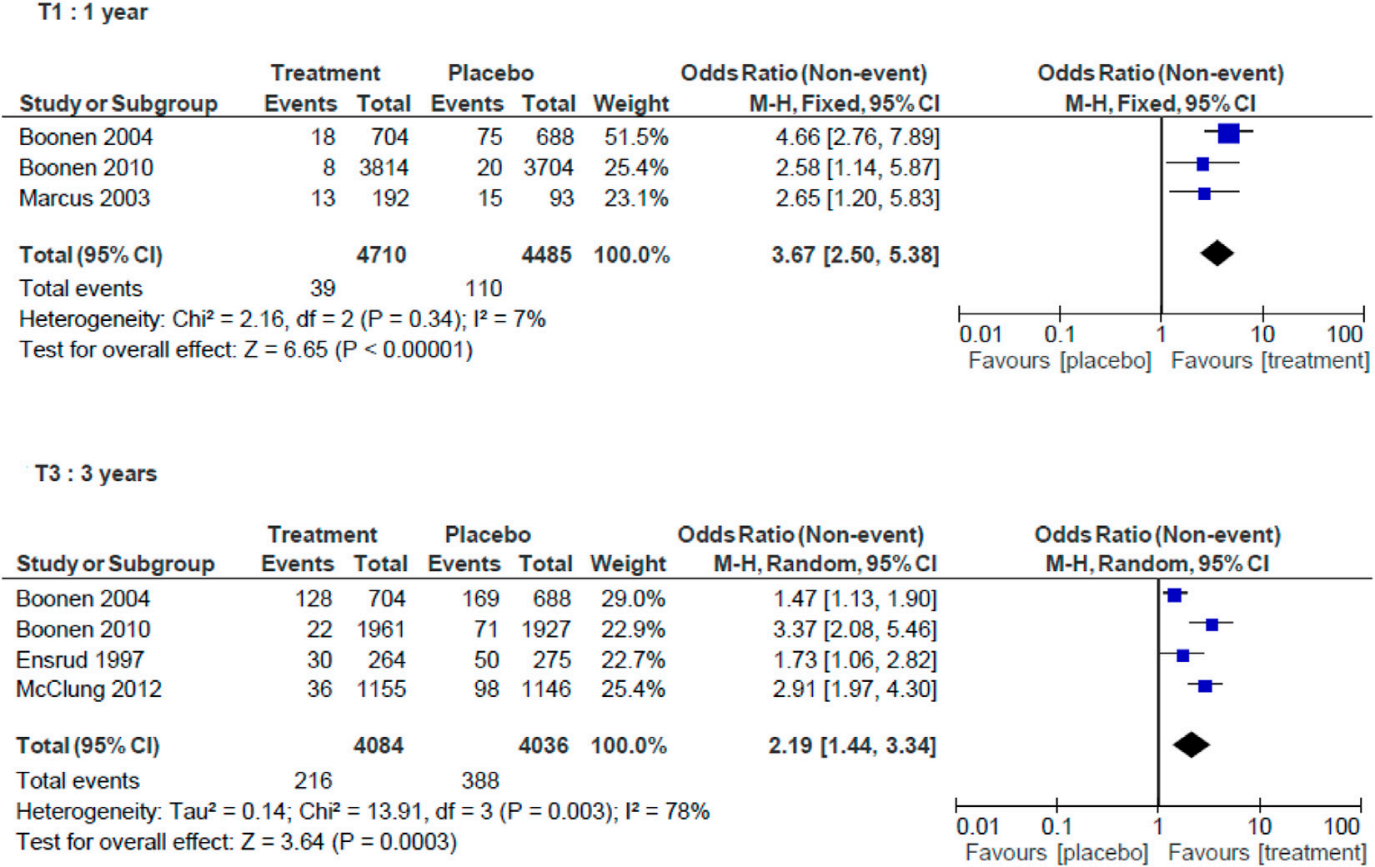
Meta-analysis of vertebral fractures.

At 1 year, analysis of data from three studies (Marcus et al., 2003; Boonen et al., 2004; Boonen et al., 2010) demonstrated that lack of treatment was associated with an increased risk of VF (OR = 3.67; 95%CI = 2.50–5.38; *p* < 0.00001). There was no evidence of statistical heterogeneity across studies (I^2^ = 7%) (Figure 3)*.*

At 3 years, analysis of data from four studies (Ensrud, 1997; Boonen et al., 2004; Boonen et al., 2010; McClung et al., 2012) showed that lack of treatment was also associated with an increased risk of VF (OR = 2.19; 95%CI = 1.44–3.34; *p* = 0.0003). There was evidence of high heterogeneity (I^2^ = 78%), so a random effect was performed (Figure 3)*.*

#### Hip fracture

**FIGURE 4:**
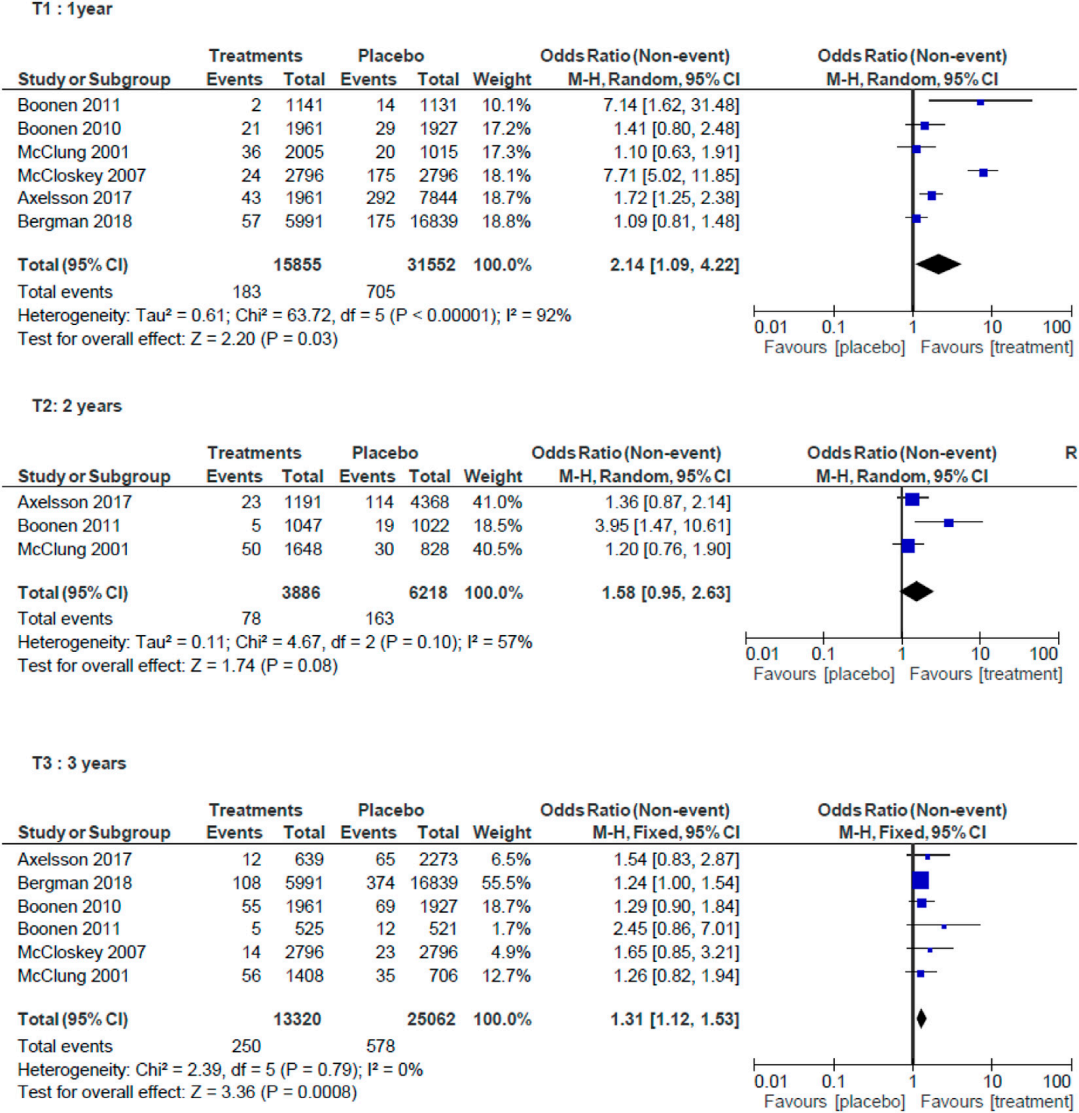
Meta-analysis of hip fractures.

At 1 year, analysis of six studies (Mcclung et al., 2001; McCloskey et al., 2006; Boonen et al., 2010; Boonen et al., 2011; Axelsson et al., 2017; Bergman et al., 2018) showed an increase in the incidence of HF (OR 2.14; 95%CI = 1.09–4.22; *p* = 0.03) in untreated subjects. A random effects meta-analysis was performed because of high heterogeneity (I^2^ = 92%) (Figure 4).

At 2 years, analysis of three studies (Mcclung et al., 2001; Boonen et al., 2011; Axelsson et al., 2017) found that lack of treatment was not associated with an increased risk of HF (OR = 1.58; 95%CI = 0.95–2.63; *p* = 0.08). Random effects meta-analysis was performed (I^2^ = 57%) (Figure 4)*.*

At 3 years, analysis of six studies (Mcclung et al., 2001; McCloskey et al., 2006; Boonen et al., 2010; Boonen et al., 2011; Axelsson et al., 2017; Bergman et al., 2018) showed an increase in the incidence of HF (OR = 1.31; 95%CI = 1.12–1.53; *p* = 0.0008) in untreated subjects. Fixed effect meta-analysis was performed (I^2^ = 0%) (Figure 4)*.*

### Sensitivity analysis

Results of sensitivity analysis were similar to the results of the study, across all criteria of the HF and VF subgroups.

## Discussion

To the best of our knowledge, this is the first systematic review and meta-analysis to examine the effectiveness of osteoporosis treatments specifically in older people. Concerning VF, most of the studies selected for systematic review showed significant results concerning fracture reduction with osteoporotic treatment (10 of 13), contrary to HF (2 of 7). This could be explained by the fact that some studies concerning HF included patients with prevalent HF, but without confirmation of established osteoporosis (McCloskey et al., 2006; Morin et al., 2007; Bawa et al., 2015; Axelsson et al., 2017; Bergman et al., 2018).

Results of the meta-analysis showed that osteoporosis treatments are associated with a reduction in the risk of VF and HF in people aged over 75 years. This is consistent with previously published reviews (Schneider, 2008; Inderjeeth et al., 2009; Vandenbroucke et al., 2017). Only the analysis of HF at 2 years found that lack of treatment was not associated with an increased risk of HF. This can be explained by the low number of studies available for analysis (3) compared to the one and 3 year analysis each comprising six studies.

In our analysis, we considered osteoporosis treatments as a whole for the treatment category in the meta-analysis because data were insufficient to envisage a separate analysis for each molecule. However, there is wide heterogeneity among the different treatments. Indeed, indication, dosage, frequency, route of administration and mechanism of action are not the same. Some drugs, such as bisphosphonates, are only antiresorptive drugs, contrary to teriparatide or abaloparatide, which are bone-forming agents, with a different mode of action. Among the bisphosphonates, some need to be taken orally every week, like alendronate, while others must be injected intravenously every year, like zoledronic acid. All these factors could influence medication adherence and consequently, efficacy, especially in older populations (Hughes, 2004).

We focused our analysis on the two most common and serious types of fractures in older people, namely VF and HF. There were not enough data to analyze other types of fracture. We chose to separate the results, because VF are often atraumatic, while HF are often due to moderate trauma, such as a fall. Indeed, in older subjects, falls are probably the strongest single risk factor in over 90% of HF (Järvinen et al., 2008). This may explain the lower efficacy of osteoporosis treatments against HF. Vitamin D supplementation was also not systematic or was insufficient in some studies, whereas it has been proven that ≥800 IU of vitamin D daily has a favorable effect in the prevention of HF in older people (Bischoff-Ferrari et al., 2012).

Medication adherence (MA) is defined as the extent to which prescribed medications are taken according to the dosage and frequency recommended by the provider (Cramer et al., 2008). It is estimated that between 30 and 50% of people do not take their medications as prescribed (Sabaté, 2003) It is therefore essential to offer multidisciplinary care to improve patient compliance and allow better effectiveness of anti-osteoporosis treatments. A list of recommendations has been issued to promote this (De Vincentis et al., 2021).

In this study, we evaluated the efficacy of osteoporosis treatments, but not safety. There were only six studies in our selection that evaluated the adverse effects specifically in older people (Boonen et al., 2004; Boonen et al., 2006; Boonen et al., 2010; Boonen et al., 2011; Greenspan et al., 2015; Axelsson et al., 2017); four of these were *post hoc* studies and therefore, their selection criteria did not enable the inclusion of older patients with multiple comorbidities and multiple medications.

Furthermore, patients over 75 years of age represented a small proportion of the overall sample in each study. In Europe and the United States of America, the annual risk of VF increases with age, from 0.4 to 0.6% in women aged 50–54 years to 1.2–1.3% between 65 and 69 years and to 2.9–3.8% after 85 years (O’Neill et al., 2009). Yet only one RCT included exclusively frail elderly women and did not observe a reduction in VF (Greenspan et al., 2015). Clinical trials will need to include more older people than previously, and should actively seek to include patients with extensive comorbidities in order to better assess the effectiveness of osteoporotic treatments in this age group.

This review has strengths and limitations that should be taken into account when interpreting the results. The strengths were that we used the well-established PRISMA process and the studies were rigorously identified *via* a double search by two independents reviewers, with the support of experienced methodologists (MP and FM) and a biostatistician (AG) to ensure the right search terms and high quality databases were used. We also improved the validity of the search by using the broadest possible search terms and considering all potential studies that covered the research topic. Despite this detailed approach, we identified only 19 publications for inclusion. Some relevant papers may have been missed due to the search strategy, the choice of databases, inconsistent search terminology, indexing problems or the filters used. In addition, we did not include gray or theoretical literature or papers that were not published in English or French.

## Conclusion

In conclusion, a reduction in the risk of VF with osteoporosis treatments was observed in most of the studies included and meta-analysis showed that lack of treatment was significantly associated with an increased risk of VF. Concerning HF, majority of included studies did not show a significant reduction in the occurrence of HF with osteoporotic treatments, but meta-analysis showed an increased risk of HF without osteoporotic treatment. Nevertheless, data are sparse concerning this age group, and most studies included were *post hoc* analyses or observational studies. Additional RCTs are thus needed to confirm the efficacy of osteoporosis treatments in reducing the risk of HF or VF in persons aged 75 years and older.

## Data Availability

The original contributions presented in the study are included in the article/Supplementary Materials, further inquiries can be directed to the corresponding author.
